# The Medullary Audio–Vocal Network in the Toad *Bombina orientalis*

**DOI:** 10.1002/cne.70088

**Published:** 2025-10-07

**Authors:** Stefan Huggenberger, Wolfgang Walkowiak

**Affiliations:** ^1^ Institute of Anatomy and Clinical Morphology Witten/Herdecke University Witten Germany; ^2^ Institute for Zoology University of Cologne Cologne Germany

**Keywords:** acoustic communication, Anura, fire‐bellied toad, isolated brain, neural analysis, single‐neuron recordings, sound generation

## Abstract

Anurans are an established paradigm to study vocal mechanisms in vertebrates. Regarding the motor patterns, airborne vocalization of most evolutionarily old anurans (Archaeobatrachia) resembles breathing—that is, lung inflation is used to generate sound. Vocal behavior and call timing can be rapidly elicited or modulated by auditory stimulation so that, for example, calls are uttered antiphonally in a chorus to avoid acoustic overlap. Accordingly, in an in vitro preparation of the isolated whole brain of the Chinese fire‐bellied toad, *Bombina orientalis*, motor patterns similar to those of respiration and vocalization can be elicited reliably by stimulation of the posterior (auditory) branchlet of the statoacoustic nerve (N. VIII). Here, we show that audio–vocal integration does not exclusively involve higher brain centers such as mesencephalic torus semicircularis (colliculus inferior) but is in parallel and more rapidly accomplished within the medulla oblongata. We recorded 228 neurons in the areas of motor nuclei of Nn. V, X, and XII. Hypoglossal motor neurons showed fast activation (latency of the first action potential: 9.9 ± 2.3 ms), exceeded by the very fast activation of interneurons within the hypoglossal area (minimum latency of action potential peak 2.9 ms) after N. VIII stimulation. These data support the idea that the area between the roots of the vagal (N. X) and hypoglossal (N. XII) nerves may be a crucial part of the breathing rhythm generator. It suggests that the hypoglossal area plays an important role in controlling the other motor nuclei involved in vocalization. Thus, this post‐vagal area may be an important supplementary interface of audio–vocal integration to initiate and coordinate vocal motor patterns and probably inhibit buccal respiration. With this information, a hypothetical motor network of buccal and lung ventilation, as well as vocalization, was constructed.

## Graphical abstract

Hypothetical network of audio‐vocal integration of motor neurons (twin circles) and interneurons (circles) in the medulla oblongata of the toad.

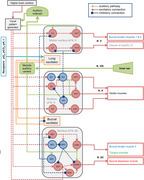

## 1 Introduction

In anuran amphibians, vocal communication plays an important role in territorial and/or mating behavior. The main call types are advertisement, release, and aggressive or clasping calls (reviewed by Kelley 2004). Since anurans are often found in large numbers during the mating season at breeding sites, males usually utter calls in groups of several animals. This leads to acoustic interaction, which influences the calling behavior of individuals (Walkowiak 1988). Because the energy consumption of calling behavior is extremely high (Taigen and Wells 1985), it is important for each animal to maximize its effectiveness. This can be achieved by adjustment of the temporal relationships between the calls within a chorus of conspecifics. Calls can be placed on top of another call, antiphonally with them, or following with either a very short or very long latency (Wells and Schwartz 2006). Among the most fascinating features in the calling behavior of frogs is the very fast vocal response to auditory signals (e.g., minimum response latencies of 55 ms in the treefrog *Smilisca sila*), raising the question of how such short latencies can be achieved (Ryan 1986). Antiphonal calling of fire‐bellied toads of the genus *Bombina* is another example of fast vocal response to auditory stimuli (Akef and Schneider 1985; Walkowiak 1988; Mohr and Schneider 1993). The short reaction time of 120–130 ms to suppress calling in *Bombina bombina* (Walkowiak 1988) demonstrates that the exact audiomotor coordination is crucial for call initiation and antiphonal calling. Mohr and Schneider (1993) and Strake (1995) reported an activation phase for calls in *Bombina orientalis* of 80–100 ms. In the túngara frog *Engystomops pustulosus* (species’ names were used according to AmphibiaWeb [2025]), this “motor delay” (Greenfield and Rand 2000) is at least 50 ms (Larter and Ryan 2024). During this phase, suppression of a following call cannot be achieved by an acoustic stimulus, and the next call is placed at the end of the activation phase. Additionally, the time between laryngeal muscle activation and the production of an audible call is approximately 40 ms (Strake 1995). Accordingly, the maximal time for interaction between the auditory and the motor system for suppression would be less than 60 ms in *B. bombina*.

Responses to acoustic or auditory input mimicking stimulation have been recorded in vivo and in vitro, respectively, with a delay of approximately 20–40 ms (minimum 5 ms) in the mesencephalic torus semicircularis (the area homologous to the human colliculus inferior) of *B. orientalis* and *Discoglossus pictus* (Walkowiak 1980; Luksch and Walkowiak 1998; Endepols and Walkowiak 2001), and the earliest responses in the thalamus of *B. bombina* could be recorded in vivo after 60–70 ms (Walkowiak 1988). These results, together with the audio–motor interaction time described above, suggest crucial connections for the initiation of calling between the auditory pathway and motor projections already on the level of the auditory midbrain or the lower brainstem. One important audio–motor interface was discovered within the torus semicircularis (Walkowiak 1992; Strake et al. 1994; Walkowiak and Luksch 1994). Moreover, the mesencephalic torus semicircularis was regarded as an audiomotor gateway between the medullary centers and the forebrain (Endepols and Walkowiak 1999, 2001; Wilczynski and Ryan 2010). Additional midbrain areas, that is, the lateral and dorsal tegmentum, were identified to be involved in audio–motor integration (Strake et al. 1994; Luksch and Walkowiak 1998; Endepols et al. 2000).

Additionally, Aitken and Capranica (1984) predicted a direct route from the auditory centers to premotor neurons within the medulla oblongata due to the short latencies (10–15 ms) of activation of medullary single units evoked by auditory stimulation of immobilized *Rana pipiens*. Thus, this route would probably bypass the auditory midbrain centers.

The respiration of *B. orientalis* and *D. pictus* functions as a pressure pump (DeJongh and Gans 1969; Gaupp 1896; Panizza 1845; Townson 1794) and can be divided into buccal oscillation and lung ventilation. During buccal oscillation, the air in the mouth cavity is exchanged via the nostrils by up and down movements of the mouth floor. During lung ventilation, air is forced from the mouth cavity into the lungs. The cycle of lung ventilation was summarized in our recent article (Huggenberger and Walkowiak 2024).

Most neobatrachian species (such as the abovementioned *R. pipiens* and *H. arborea*) generate their calls using the expiratory air stream. The air is forced from the lungs through the larynx into the buccal cavity and, if present, the vocal sacs by means of contraction of the trunk musculature (Martin and Gans 1972; Girgenrath and Marsh 1997). In contrast, the archaeobatrachian species mentioned above call without activation of the trunk muscles (Strake 1995). *Discoglossus pictus* emits calls during both inspiration and expiration (Strake 1995), and all members of the genus *Bombina* use only the inspiratory air stream to produce calls with the lungs as functional resonators (Lörcher 1969; Strake et al. 1994; Walkowiak 1992; Walkowiak 2007). The muscle activity pattern during vocalization in these inspiratory calling species is largely identical with the pattern of lung inflations but with a prolonged inspiration phase (Strake 1995; Walkowiak 2007; Huggenberger and Walkowiak 2024).

The motor system responsible for inspiratory call generation, that is, the buccal and gular regions, has multiple functions: feeding, respiration, and vocalization (DeJong and Gans 1969; Martin and Gans 1972; Nishikawa and Roth 1991). Accordingly, vocal generation in *Bombina* spec.—as is the case in most vocalizing terrestrial vertebrates—must be coordinated with the lung ventilation rhythm while buccal oscillations (used to exchange breathing gases via the buccal mucosa), mouth movements for prey capture, and feeding would be inhibited.

According to the coordinated pattern of muscle activation needed for respiration and vocalization, corresponding activity can be found in the relevant nerves. Huggenberger and Walkowiak (2024) analyzed the fictive lung ventilation and vocalization behavior of isolated brains of *B. orientalis* by nerve root recordings. After stimulation of N. VIII, the “buccal depressor nerve” (N. XII) was activated (termed *phase A*). Afterward, the laryngeal branch of N. X and the “buccal elevator nerves” were active (termed *phase B*). In the following phase (*phase C*), the “buccal depressor nerve” may be active again. Thus, these activation patterns resembled buccal dilatation (*phase A*) to fill the mouth cavity with air. Then, buccal depression (*phase B*) followed for lung inspiration. After this *phase B*, there may follow the next buccal dilatation (*phase C*) for lung expiration and to fill the mouth cavity with fresh air through the nostrils. Moreover, we (Huggenberger and Walkowiak 2024) were able to distinguish between fictive respiration and fictive advertisement call generation of isolated brains of male *B. orientalis*. As described above, both movement patterns and, thus, both neural patterns resembled each other. However, male's advertisement calls in vivo had the same general pattern compared to lung ventilation but longer duration periods (Strake 1995; Walkowiak 2007). Accordingly, male brains in vitro showed increased durations of motor nerve activities after electrical stimulation of N. VIII in comparison to female brains. These prolonged activities could be interpreted as fictive calling (Huggenberger and Walkowiak 2024). The increased durations were also elicited in male brains by stimulation of vocalization‐relevant forebrain structures such as the preoptic area but not during spontaneous activities.

Vocal premotor areas in anurans were described by Schmidt (1992) as two separate semi‐independent pattern generators, located in the brainstem of *R. pipiens*. The first pattern generator is located just anterior to the motor nucleus of the trigeminal nerve in the medulla and hence was named pretrigeminal nucleus (preV; Schmidt 1976) or dorsal tegmental area of the medulla (DTAM; Kelley 1980; Wetzel et al. 1985; Zornik and Kelley 2007, 2008). This brain region (preV) may be involved in vocalization since it projects into relevant motor areas, that is, cranial nerve nucleus IX–X, which is homologous to the nucleus ambiguus in mammals (Wetzel et al. 1985; Brahic and Kelley 2003; Albersheim‐Carter et al. 2016; Barkan and Zornik 2020; Kelley 2022). Zornik and Kelley (2008) demonstrated that neurons of the preV may coordinate and produce vocal motor output by directly activating laryngeal motor neurons of the nuclei IX–X in *Xenopus laevis*. However, it must be kept in mind that in the fully aquatic *X. laevis*, as a member of the Pipidae, only intrinsic laryngeal musculature is active during calling (Kelley 1980; Brahic and Kelley 2003; Kelley et al. 2020) without the need for coordination of larynx movements and lung ventilation as in terrestrial frogs and most other tetrapods (Kelley 2022). Nevertheless, the interpretations deriving from results gathered in *X. laevis* may be true for many vertebrates since the preV may be homologous to the mammalian parabrachial nucleus (including the subparabrachial Kölliker–Fuse nucleus). This nucleus is a crucial part of vocal control in mammals (Jürgens 2009; Smotherman et al. 2006; Kelley et al. 2020; Barkan and Zornik 2020; Barkan et al. 2021; Schröder et al. 2020; Kelley 2022).

The second pattern generator, described as the classical pulmonary respiration generator, was found in the region of the motor nuclei IX–X using lesion experiments in isolated brains of *R. pipiens* (Schmidt 1992). However, damage to motor nuclei IX–X eliminated normal neural correlates of vocal activity in preV (Schmidt 1976, 1992), which indicated that the preV requires reciprocal input from the motor nuclei IX–X to generate proper vocal patterns. Based on these findings, Schmidt (1992) proposed a model in which circuits in the motor nuclei IX–X produce respiratory patterns, whereas the interactions between preV and motor nuclei IX–X are responsible for producing vocal patterns in *R. pipiens*.

### 1.1 Aim of This Study

Using an in vitro preparation of the isolated brain of *B. orientalis*, we examined the influence of auditory input on vocal premotor and motor centers within the anuran medulla oblongata. In the isolated brain, motor patterns like those of lung ventilation can be elicited by electrical stimulation of the auditory branchlet of the N. VIII (Huggenberger and Walkowiak 2024). Using this technique, we examined the mechanisms of motor pattern generation and its modulation by intracellular recordings in motor areas of buccal depressor and levator muscles. Here, we follow the following questions:

- Where in these motor areas arrives the auditory information and when which quality (excitatory and inhibitory)?
- What target neuron types are involved in audiomotor integration within the medullary motor network?
- How buccal oscillations and voiceless lung ventilations may be inhibited?

We used this information for the construction of a putative motor network of buccal and lung ventilation as well as vocalization.

## 2 Material and Methods

### 2.1 Neurograms

**FIGURE 1:**
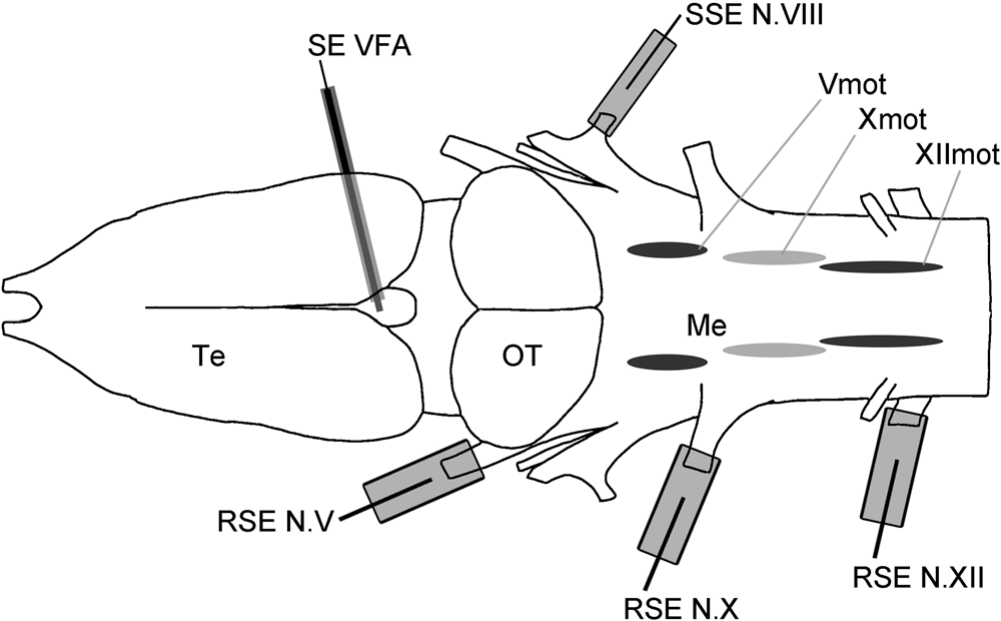
Schematic representation of an isolated *Bombina orientalis* brain in dorsal view (rostral pointing left) showing the recording sites at Nn. V, X, and XII contralaterally to the stimulation site at N. VIII. The intracellular recording sites in the area of the motor nuclei of the respective nerves (Vmot, Xmot, XIImot) were situated contralaterally to the stimulation at N. VIII. Me, medulla; OT, optic tectum; RSE, recording suction electrode; SSE, stimulus suction electrode; Te, telencephalon.

All experiments were conducted on 13 female and 33 male adult Chinese fire‐bellied toads, *B. orientalis*, from the breeding stock of the Department of Zoology (Biocenter, University of Cologne, Germany). Isolated brain preparations were carried out using the methods described by Luksch et al. (1996). In short, animals were deeply anesthetized with 0.2% tricaine methanesulfonate solution (MS 222; Sigma–Aldrich, Poole, UK) in tap water (pH 7.4, adjusted with NaHCO_3_) for 10 min and cooled in ice to a body temperature of approximately 5°C. The animals were perfused transcardially with 10–20 mL of iced Ringer solution (104 mM NaCl, 25 mM NaHCO_3_, 2.4 mM CaCl_2_, 4 mM KCl, 1.4 mM MgCl_2_, 11 mM glucose) that had been oxygenized with carbogen (95% O_2_, 5% CO_2_) to a pH of 7.3–7.6. The complete brain and spinal cord were isolated by a ventral approach. The nerves were cut with microscissors, and special care was taken with the cranial nerves V, VIII, X, and XII. Afterward, the pituitary gland, the dura mater, and the choroid plexus were removed from the isolated brain. The brain was then kept overnight in 400 mL of freshly oxygenized Ringer solution at 4°C. The next day, the brain was transferred dorsal‐facing top into a submerged‐type recording chamber and perfused continuously with freshly oxygenized Ringer solution at 16°C (>2 mL/min; Warner Instruments Temperature Controller, Hamden, CT, USA). To record compound potentials (spontaneous and stimulus‐driven activity) of the motor output of Nn. V, X, and XII, custom‐made suction electrodes were used (Figure 1; Huggenberger and Walkowiak 2024) with a band‐pass‐filtered AC amplifier (100–1000 Hz; A‐M Systems Differential AC Amplifier Model 1700, Sequim, WA, USA). Electrical stimuli (single pulses and pulse trains of five, 10, or 20 pulses with a frequency of 10, 20, or 40 Hz, respectively) were applied to the posterior (auditory) branchlet of N. VIII via a custom‐made suction electrode and a stimulus isolator (ISO Flex, AMPI) triggered by a pulse generator (Master 8, AMPI, Jerusalem, Israel).

### 2.2 Single‐Neuron Recordings

Simultaneously with the recordings of the compound potentials of the isolated brains, intracellular recordings of single neurons in the areas of the motor nuclei of Nn. V, X, and XII (Vmot, Xmot, XIImot) were conducted using sharp microelectrodes. The electrodes were pulled from filamented capillaries (Hilgenberg, Malsfeld, Germany, 1.5 mm outer diameter, 0.315 mm wall thickness) with a Brown Flaming puller (Sutter Instruments, Novato, CA, USA) to impedances of 100–160 MΩ when filled with 3 M potassium acetate and 100 mM KCl. Electrodes were connected to a current clamp intracellular amplifier (Cyto 721, WPI, Sarasota, FL, USA).

The sharp microelectrode was placed vertically above the brain. Using the nerve roots as landmarks, Vmot, Xmot, or XIImot were approached dorsally in the half of the medulla oblongata ipsilateral to the stimulated N. VIII. The motor nuclei are situated medially and deep to the sulcus limitans of His, thus near the midline (Nieuwenhuys and Opdam 1976; ten Donkelaar 1998; Endepols and Walkowiak 2001). Vmot could be reached exactly caudal to the cerebellum.

Recordings were digitized using a data acquisition unit (Micro 1401, CED, Cambridge, UK) and the corresponding software (Spike 2, CED, Cambridge, UK) and evaluated off‐line using Spike 2. Mann–Whitney *U*‐tests were used to test for significant differences using SPSS 19.0 (SPSS Inc., IBM Inc., Armonk, NY, USA). If not stated otherwise, the statistical means ± *SD* are given throughout the text.

Motor neurons were defined by coincident action potentials (APs) in the compound recording of the respective nerve. If these coincident APs failed, the neuron was regarded as an interneuron. Both motor neurons and interneurons were classified into types due to their de‐ or hyperpolarization responses during the phases of compound activity (see Section 3 and Huggenberger and Walkowiak 2024).

Some of the neurons were filled with biotin ethylenediamine (Neurobiotin, Molecular Probes, Eugene, OR, USA) after the recordings. For these recordings, the microelectrodes were filled with 3% Neurobiotin in 0.3 M K^+^‐acetate and 100 mM KCl, and positive current (2.5 nA) was applied for 10 min via the microelectrode. Thereafter, the brains were kept at least 1 h in the recording chamber as described above. Then, the brains were fixed with 4% paraformaldehyde and 1.25% glutaraldehyde in 0.1 M sodium phosphate buffer (PB; pH 7.4) overnight. After washing in PB, brains were embedded in 4% agar (Merck, Darmstadt, Germany) in PB and cut into 50‐µm transverse sections using a Vibratome (Leica VT 1200S, Wetzlar, Germany). Sections were directly mounted onto chrome alum/gelatin‐coated slides, dried at 37°C, rinsed in PB for 10 min, and incubated with 2% streptavidin–horseradish peroxidase (HRP; Amersham, Braunschweig, Germany) + 0.5% Triton‐X 100 (Serva, Heidelberg, Germany) in PB overnight. After several washes in PB, HRP was visualized by using a reaction with DAB (Boeringer, Mannheim, Germany) and heavy metal intensification (Adams 1981). H_2_O_2_ was provided by a glucose oxidase reaction (Shu et al. 1988). After staining, sections were dehydrated in ethanol and coverslipped with Corbit (Hecht, Kiel‐Hassee, Germany). For further information about the staining procedure, see Roden et al. (2005). Labeled neurons were manually reconstructed with the aid of a drawing tube mounted onto a microscope (Leitz, Orthoplan) at a magnification of x200.

## 3 Results

**FIGURE 2:**
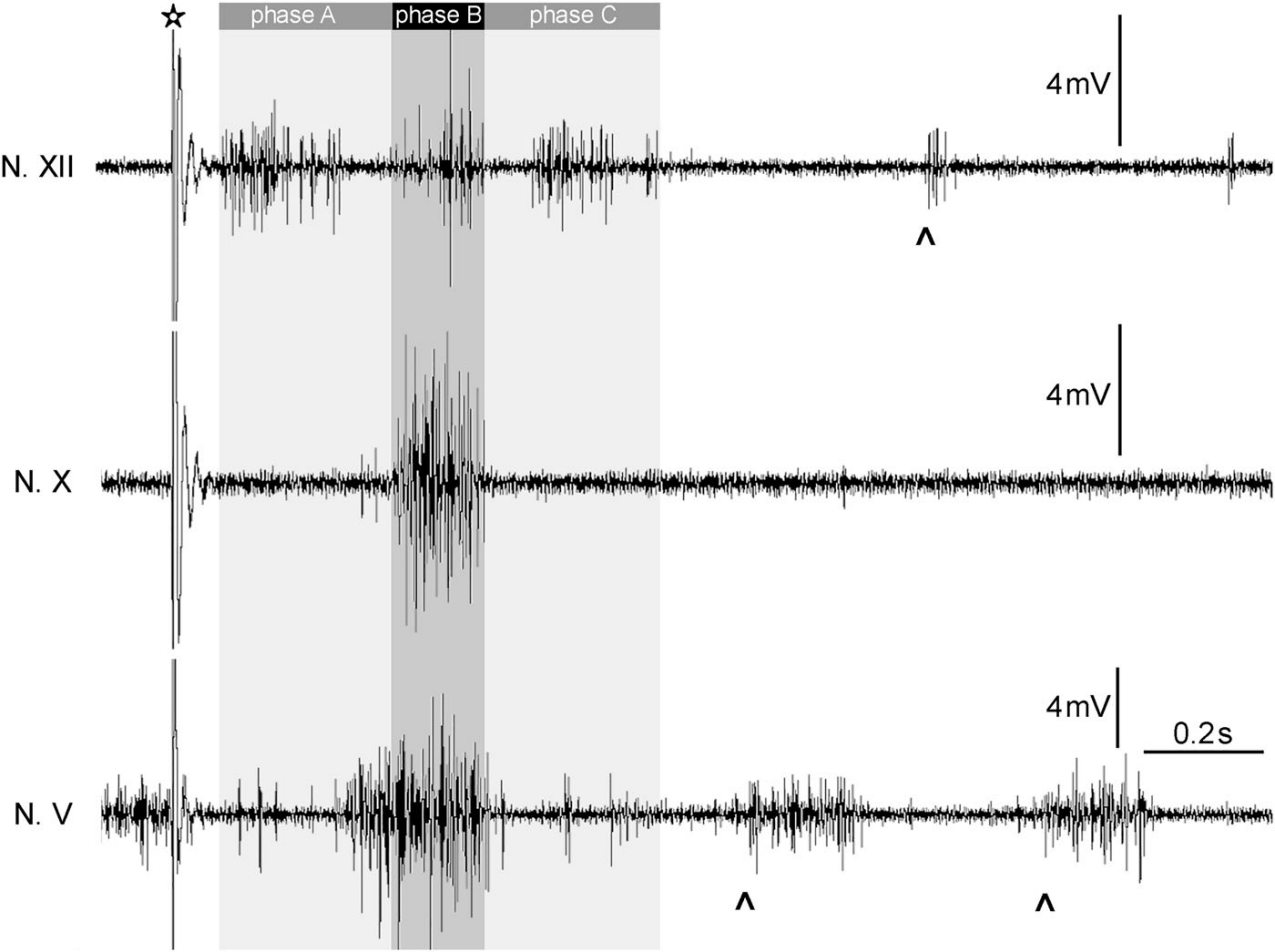
Representative compound potential recordings at Nn. V, X, and XII in the isolated brain of *Bombina orientalis*. The artifact elicited by stimulation of N. VIII is indicated by the asterisks. The typical motor pattern after auditory nerve stimulation starts with the activation of N. XII motor neurons (*phase A*). Thereon, the second activation phase (*phase B*) was characterized by high activities of the three nerves recorded. Note that the burst of N. V starts in advance of *phase B*. During *phase C*, only N. XII is active except for the slightly prolonged activity of N. X. The upward arrow heads indicate the spontaneous alternating low‐amplitude activation of Nn. V and XII, which may follow the stimulus‐driven compound activations representing fictive buccal oscillations. Further details to characterize the phases of nerve activity can be found in Huggenberger and Walkowiak (2024).

Spontaneous and stimulus‐driven activity of single motor neurons and interneurons (*n* = 233) was recorded intracellularly within the area of motor nuclei of Nn. V, X, and XII. In parallel, neurogram compound potentials of Nn. V, X, and XII were recorded (Figure 2). For a detailed description of these neurograms, see Huggenberger and Walkowiak (2024). Motor neurons were defined by coincident APs in the compound recording of the respective nerve. To further divide neuron types, neurogram compound recordings (spontaneous and after stimulation of N. VIII) representing fictive lung inflations (cf. Kogo et al. 1994; Huggenberger and Walkowiak 2024) were divided into three successive phases. The first activation (*phase A*; Figure 2) was characterized by a high‐amplitude burst of N XII. During this phase, Nn. V and X were less active. Thereon, the second activation phase (*phase B*; Figure 2) was characterized by highly coincident activities of the three nerves recorded. The *phase C* (Figure 2) followed next with a burst of N. XII.

Additionally, spontaneous low‐amplitude activations were recorded, which resembled fictive buccal oscillations (cf. Kogo et al. 1994; Huggenberger and Walkowiak 2024), during which N. X was not active. The fictive buccal oscillations were characterized by alternating activations of Nn. V and XII (Figure 2).

In sum, we recorded 228 neurons intracellularly, and 127 of these were defined as motor neurons. As expected from the similarities in compound nerve potentials of fictive lung and buccal inflations (Huggenberger and Walkowiak 2024), the neuron types did not differ between females and males and between the spontaneous activation and responses after stimulation. Thus, the data were pooled.

### 3.1 Motor Neurons

**FIGURE 3:**
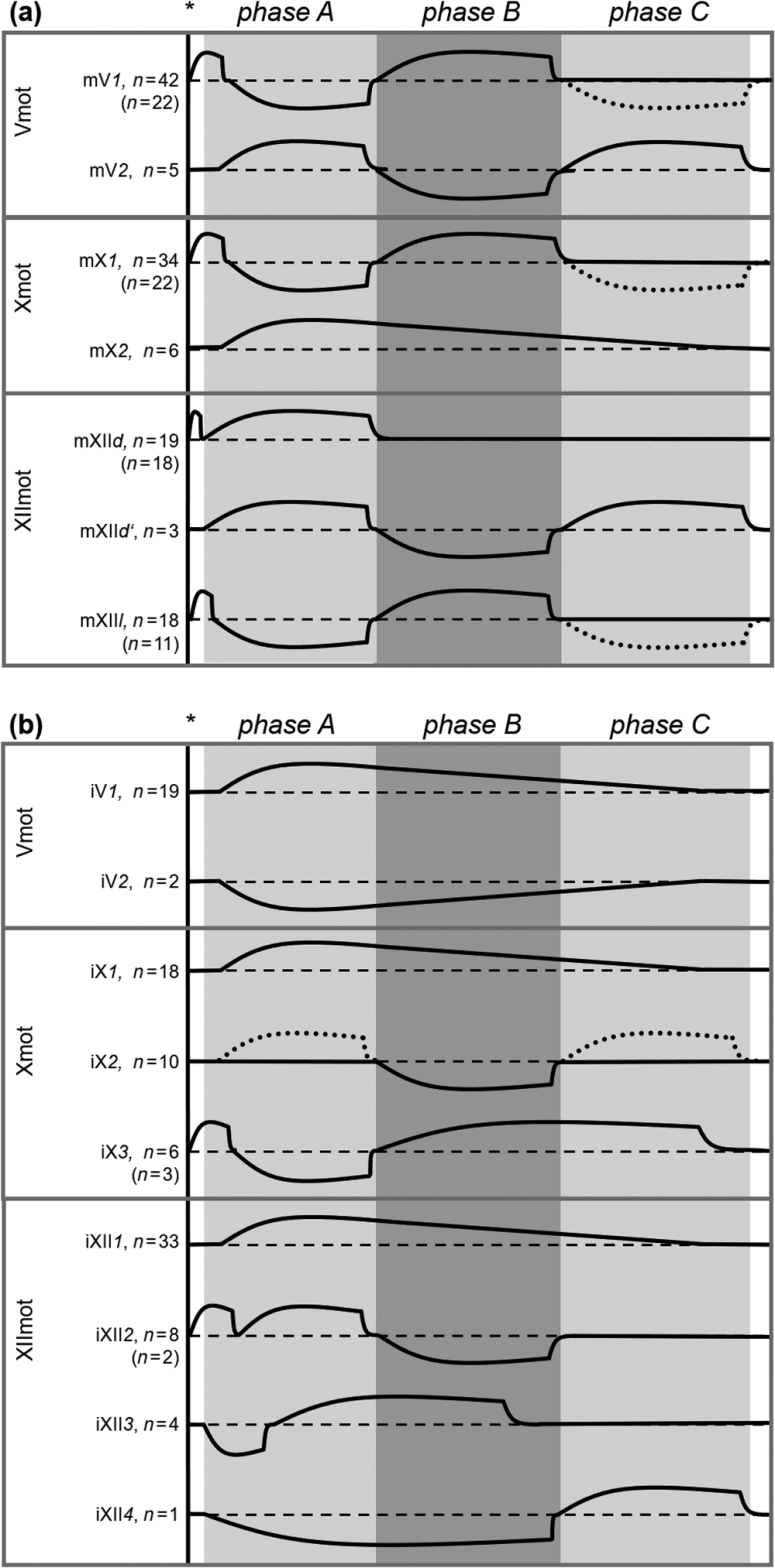
Schematic representation of voltage traces (voltage over time) of response types of motor neurons (a) and interneurons (b) recorded in the areas Vmot, Xmot, and XIImot. The gray areas represent the borders of the activation phases described in Figure 2. The solid black vertical lines represent the stimulus onsets marked by asterisks. The horizontal broken lines represent the resting membrane potential (RMP) of each neuron type as indicated. Solid lines above RMP represent excitatory postsynaptic potentials (EPSPs; action potentials were not depicted); below RMP are inhibitory postsynaptic potentials (IPSPs). The dotted lines represent EPSPs and IPSPs, respectively, that did not occur regularly in the respective types of neurons. Note the short EPSPs that indicate the partially elicited “fast EPSP” and “fast APs” induced immediately after the N. VIII stimulation. This fast input is strictly sensory since these activations cannot be observed in spontaneous fictive lung inflations (Figure 5a). The numbers of each neuron type are given in the left column; in parentheses are the numbers of neurons with additional “fast EPSP.”

After stimulation of N. VIII, we found seven different motor neuron types: two types in each area of the motor nuclei of Nn. V and X (Vmot and Xmot, respectively), and three types in the area of the motor nucleus of N. XII (XIImot; Figure 3a).

**FIGURE 4:**
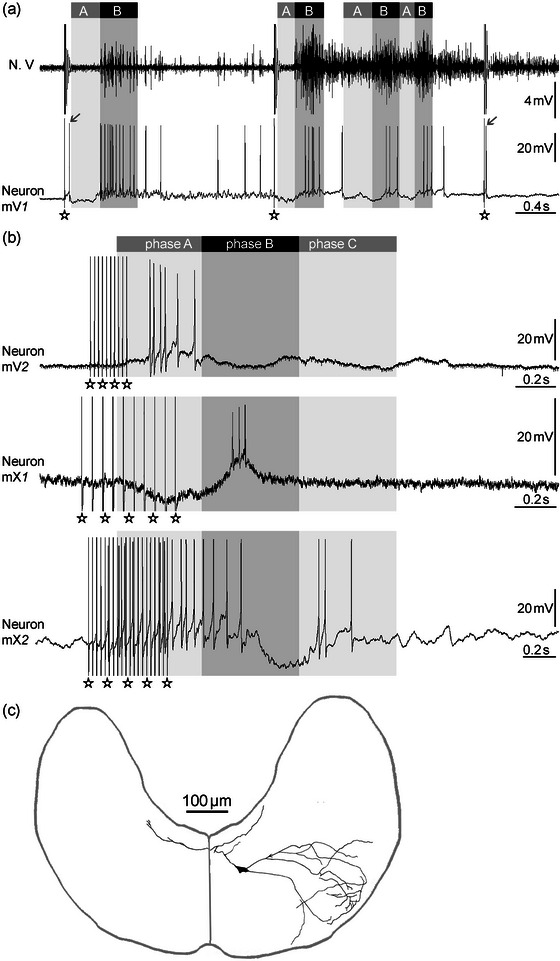
Recordings of different motor neuron types in Vmot and Xmot as indicated. The asterisks indicate the artifacts elicited by N. VIII stimulation. (a) Recordings of an mV*1* motor neuron with corresponding compound potentials of N. V (upper trace, phases A and B as indicated; *phase C* was not defined due to the extended activity of N. V). Note the “fast AP” with short delay to the first and third stimulus prior to IPSP (gray arrows). (b) Recordings of mV*2*, mX*1*, and mX*2* motor neurons as indicated. The phases of activity of Nn. V, X, and XII are indicated by the upper bars. Note that there are no “fast EPSPs.” (c) Camera lucida drawing of the mX*1* neuron shown in (b) filled by Neurobiotin with its position within a schematic transverse section. The rostrocaudal extension of this neuron was approximately 200 µm.

In Vmot, we recorded 47 motor neurons, and 42 of these (89.4%), referred to as mV*1*, fired coincidently with the main activity of the nerve in *phase B*. These mV*1* neurons became hyperpolarized during *phase A* (Figures 3a and 4). In the very early beginning of *phase A*, the mV*1* neurons became shortly depolarized (duration 112 ± 101 ms; “fast EPSP”); 22.5% of those neurons generated an AP during this “fast EPSP” with a latency of 36 ± 9 ms, which we refer to as “fast AP” (Figure 6). The “fast AP” could not be traced in the compound recordings because they are hidden by the prolonged stimulus artifacts of these compound recordings. Eleven of the mV*1* neurons were additionally hyperpolarized in *phase C*, while the other mV*1* neurons stayed silent. The second type (mV*2*, *n* = 5) is characterized by AP firing in *phase A*, hyperpolarization in *phase B*, and depolarization (with sporadic generation of APs) in *phase C* (Figure 4b).

In Xmot, two different types of motor neurons were differentiated as well (Figure 3a). Most of the neurons (32 neurons, 85%), which we refer to as mX*1*, showed a hyperpolarization in *phase A* and a depolarization (with APs) in *phase B* (Figure 4b). Three of these mX*1* neurons were additionally hyperpolarized in *phase C*. According to this pattern of postsynaptic potentials and AP firing, mX*1* neurons are very similar to the mV*1* neurons, including the short and distinct “fast EPSP” (duration 126 ± 122 ms, in 26.9% with “fast AP” generation) prior to and at the beginning of *phase A* (119 ± 41 ms; Figure 6). In comparison, mX*2* neurons were only depolarized in *phases A* and *B* but fired mainly in *phase A* (Figure 4b). Two of the six mX*2* neurons stayed depolarized in *phase C*.

**FIGURE 5:**
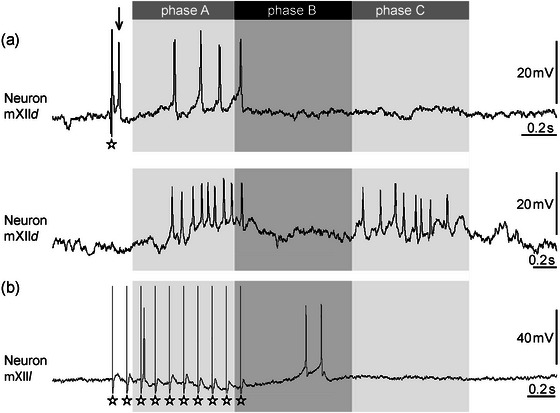
Recordings of different motor neuron types in XIImot as indicated. The phases of activity of Nn. V, X, and XII are indicated by the upper bars. The asterisks indicate the artifacts elicited by N. VIII stimulation. (a) Recordings of an mXII*d* neuron. The upper trace shows the activity of the neuron after N. VIII stimulation and the lower trace its spontaneous activation. Note the first AP (“fast AP”) marked by the arrow, which occurred prior to the slow EPSP (with additional APs) in *phase A* after N. VIII stimulation. A corresponding “fast AP” fails during the spontaneous activation. (b) Recording of an mXII*l* motor neuron. Note the short EPSPs (“fast EPSPs”) with short delays after each stimulus pulse within the long‐lasting IPSP in *phase A*.

**FIGURE 6:**
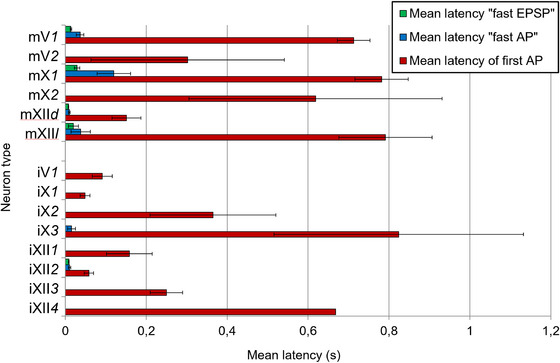
Mean latencies and SEM of neuron response types. Red bars represent the mean latency of the first AP of the motor pattern–related activity of the neuron within the high‐amplitude burst of the respective nerve (Mean latency of first AP). Blue bars represent the mean latency of the “fast AP” elicited immediately by the N. VIII stimulation (Mean latency “fast AP”). Green bars represent the mean latency of the onset of the “fast EPSP” elicited immediately by the N. VIII stimulation (Mean latency “fast EPSP”).

In XIImot, three different motor neuron types were distinguished (Figure 3a). The main type (*n* = 22) was characterized by a slow depolarization in *phase A*. This type could be subdivided in a prevalent type, referred to as mXII*d* (*n* = 19, 47.5%; the *d* stands for depressor, the putative muscle group of the neuron type, see Section 4), which shows a depolarization during *phase A* with a single AP (“fast AP”) in advance to the slow *phase A* depolarization (Figure 5a); and a rare type (mXII*d*’, *n* = 3, 7.5%) without this “fast AP” but with a hyperpolarization in *phase B* and a slow depolarization in *phase C* (not shown as voltage traces in figures due to its rarity). The latencies of the “fast AP” of mXII*d* neurons to N. VIII stimulation were the shortest found in this study (mean 9.9 ± 2.3 ms, minimum latency 4.3 ms; Figure 6) and thus significantly shorter than the “fast APs” in the other neuron types (*p* < 0.02). A second motor neuron type in XIImot, referred to as mXII*l* (*n* = 18, 45%; the *l* stands for levator, the putative muscle group of the neuron type, see Section 4), shows the same pattern as mV*1* and mX*1* (Figures 3a and 5b). The duration of the “fast EPSP” at the beginning of *phase A* was 77 ± 107 ms in these mXII*l* neurons (latency of “fast AP” 38 ± 24 ms; Figure 6).

The comparison of the spontaneous activity of the motor neurons with their stimulus‐elicited responses revealed that, in general, the neurons were active during the same phases of fictive lung inflations. Additionally, all motor neurons were active in both spontaneous and stimulated events. However, the spontaneous activity lacked the “fast AP” and the corresponding “fast EPSP.” Accordingly, during spontaneous activation, mV*1*, mX*1*, and mXII*l* start with a hyperpolarization in *phase A* (not shown in figures to avoid replications). mXII*d* neurons show only the slow depolarization in *phase A* and no “fast AP” in advance of *phase A* (Figure 5a). No further differences of the spontaneous firing pattern in comparison to N. XIII stimulation were found. Additionally, all motor neurons of Nn. V and XII showed antiphasic activity during spontaneous fictive buccal oscillations.

### 3.2 Interneurons

In addition to these motor neurons, we found nine different interneuron types, two in Vmot, three in Xmot, and four in XIImot (Figure 3b). In general, all of these interneurons were active spontaneously as well as after N. VIII stimulation (except the fast excitation in advance of *phase A*, see below). Of the 101 interneurons, 27 were active spontaneously in between the phases defined here.

**FIGURE 7:**
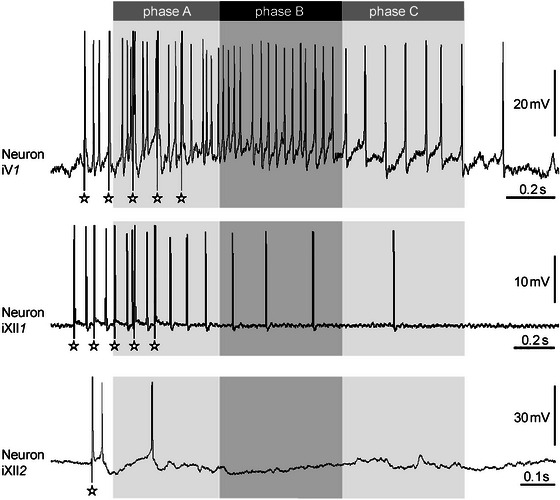
Recordings of most prevalent interneuron types (as indicated). The iX*1* neuron is not shown due to its similarity with the iV*1* and iXII*1* neurons. The phases of activity of Nn. V, X, and XII are indicated by the upper bars. The asterisks indicate the artifacts elicited by N. VIII stimulation.

In the area of Vmot, the most abundant interneuron type, referred to as iV*1* (*n* = 19; Figure 7), was depolarized in *phase A*. This depolarization could stop before *phase B* (*n* = 11), reach into *phase B* (*n* = 4), or continue into *phases B* and *C* (*n* = 4). These iV*1* interneurons had a mean latency of their first AP of 272 ± 276 ms, and the shortest latency was 12.6 ms. Only two neurons of the second type were found in Vmot (iV*2*, not shown as recorded traces in figures due to its rarity), which were hyperpolarized either in *phase A* or in all three phases.

In the area of Xmot, three different interneuron types were distinguishable (Figure 3b). The most abundant type (iX*1*, *n* = 18) showed the same pattern as iV*1* (not shown as recorded traces in figures to avoid replications). However, in contrast to the iV*1* neurons, most of the iX*1* neurons (*n* = 13) were depolarized during all three phases; three of the iX*1* neurons were depolarized during *phases A* and *B* and two of them in *phase A* only. The second interneuron type in Xmot (iX*2*, *n* = 10, not shown as recorded traces in figures) was characterized by a hyperpolarization in *phase B*. Some of these neurons were depolarized additionally in *phase A* (*n* = 1), in *phase C* (*n* = 1), or in both *phases A* and *C* (*n* = 5). The third interneuron type in Xmot (iX*3*, *n* = 6) was defined by a hyperpolarization in *phase A* and a depolarization in *phase B*. For four of these iX*3* neurons, this depolarization reached into *phase C*. Two of the iX*3* neurons showed additionally a short, distinct depolarization (“fast EPSP,” duration 284 ± 240 ms) at the beginning of *phase A* (including a “fast AP”). In general, these Xmot interneurons showed a latency of their first AP of 340 ± 86 ms, and the shortest latency of an iX*1* neuron was 4.4 ms.

In area XIImot, we found four different interneuron types (Figure 3b). Most abundant was the type referred to as iXII*1* (*n* = 33; Figure 7), which was similar to iV*1* and iX*1*. Twenty‐five of the iXII*1* neurons were depolarized only in *phase A*, six in *phases A* and *B*, and two continuously in *phases A*, *B*, and *C*. The second interneuron type in XIImot (iXII*2*, *n* = 8; Figure 7) was characterized by a depolarization in *phase A* and a hyperpolarization in *phase B* (Figure 5c). In contrast, four neurons (iXII*3*, not shown as recorded traces in figures due to its rarity) started with a hyperpolarization in *phase A*, followed by a depolarization in the same phase that could last into *phase B* (*n* = 2). A single neuron (iXII*4*, not shown as recorded traces in figures) was found that showed a hyperpolarization in *phases A* and *B* and a depolarization in *phase C*. The AP latency of these XIImot interneurons, in general, was short (186 ± 47 ms), and the shortest latency was 2.9 ms and 3.0 ms, respectively, found in two iXII*2* interneurons that revealed a “fast AP” in advance of the slow depolarization of *phase A* (Figure 3b).

## 4 Discussion

### 4.1 Identification of Motor Neuron Types in the Medullary Vocal Circuit

In general, we recorded seven main types of motor neurons associated with nerves’ activity pattern of lung inflation and vocalization. Corresponding to nerve activity during fictive vocalization after N. VIII stimulation, we differentiated motor neuron types that depolarized either during *phases A* and *C* or during *phase B* (Figure 3). In comparison to the myograms of Strake (Strake et al. 1994; Strake 1995) and the neurograms of our previous study (Huggenberger and Walkowiak 2024), it is likely that the neuron types mV*1* and mXII*l* may activate the levator muscles of the mouth floor during *phase B* (hence the “*l*” for levator in our abbreviation of this neuron type), while mX*1* neurons may activate the laryngeal musculature to open the glottis in parallel. During *phase A*, mXII*d* neurons may be responsible for mouth floor depression (hence the “*d*”). The fact that all motor neurons were active spontaneously during their respective phases, as well as after N. VIII stimulation, shows that the same pools of motor neurons are active for respiration and vocalization alike (for discussion of the similarities and the differentiation between fictive respiration and fictive call generation in *B. orientalis*, see Huggenberger and Walkowiak [2024]).

The exact target of mV*2* and mX*2* neurons, which are depolarized in parallel to mXII*d* neurons, has not been identified so far. However, the firing pattern of these neurons demonstrates that the sporadic low‐amplitude activity of Nn. V and X in *phase A* (Figure 2) is elicited by the small and distinct motor neuron populations mV*2* and mX*2*. We speculated in our previous article (Huggenberger and Walkowiak 2024) that the low‐amplitude activation of N. V during *phase A* may activate the anterior muscles of the mouth floor (anterior intermandibular muscle), which closes the nares. The activation of N. X during *phase A* may be interpreted as activation of muscles that open the glottis (dilatator laryngis muscle) to fill the buccal cavity additionally with air from the lungs, as is also the case in a series of ventilatory cycles when air is sucked from the lungs into the buccal cavity.

### 4.2 Identification of Interneuron Types in the Medullary Vocal Circuit

The firing pattern of interneurons was not as clearly restricted to each phase of compound nerve activity as shown above for the motor neurons (Figure 3). However, it has to be mentioned that we cannot be sure that interneurons were located exactly within or close to motor nuclei. Due to the experimental procedure, it may be possible that some interneurons lie in the area topographically related, for example, in the adjacent reticular formation. Accordingly, we cannot verify that the interneurons identified in this study were involved in audio–vocal integration, but they may also be involved in multisensory processing or other motor tasks. However, this study demonstrates that interneurons, which get fast auditory input, are distributed throughout the complete area of the vocal motor nuclei. The shortest latencies (minimum 4.4 and 2.9 ms) were found in the areas of Xmot and XIImot, respectively.

Some of the interneuron types in area XIImot were very similar to motor neuron types of the same region (iXII*1*, iXII*2*; Figure 7c). At least in some of these cases, it is possible that these interneurons may be actual motor neurons for which we did not observe coincident APs in the compound potential recordings, for example, due to insufficient sealing of the suction electrode or destroyed branchlets of the thin N. XII.

### 4.3 Hypothetical Network of Audio–Vocal Integration

We used the information on the electrophysiological responses of motor neurons and interneuron types and their locations within the medulla oblongata to model the connection patterns of a hypothetical network of audio–vocal integration. We integrated the information of auditory input and respiratory oscillators (references above) and our results presented here into a network by plausibility, considering the motor pattern output.

As shown in our recent article (Huggenberger and Walkowiak 2024), the same network is used for lung ventilation and vocalization. Thus, we cannot distinguish the output pattern (compound potentials), except for the length of *phase B* in males after N. VIII stimulation (Huggenberger and Walkowiak 2024). Since both motor neurons and interneurons did not show significant differences between their spontaneous activity and after stimulation, we conclude that the network components of lung ventilation and vocalization share the same neural components.

In general, fast medullary input and midbrain input from torus semicircularis activate premotor interneurons as well as motor neurons (Walkowiak 1980; Luksch and Walkowiak 1998; Endepols and Walkowiak 2001; Wilczynski and Endepols 2007). According to our data presented here, this leads to short‐latency activation of depressor motor neurons (mXII*d*) in *phase A*. The activity of levator motor neurons must be initially suppressed in parallel via fast inhibitory interneurons, for example, by iXII*2* neurons (Figure 3b). Then, during *phase B*, depressor activity must be terminated, probably by delayed firing inhibitor interneurons such as iXII*3* neurons (Figure 3b). Simultaneously, levator units (mV*1*, mX*1*, mXII*l*) must be activated by interneurons such as iV*1*, iX*1*, iX*3*, and iXII*1* (Figure 3b).

**FIGURE 8:**
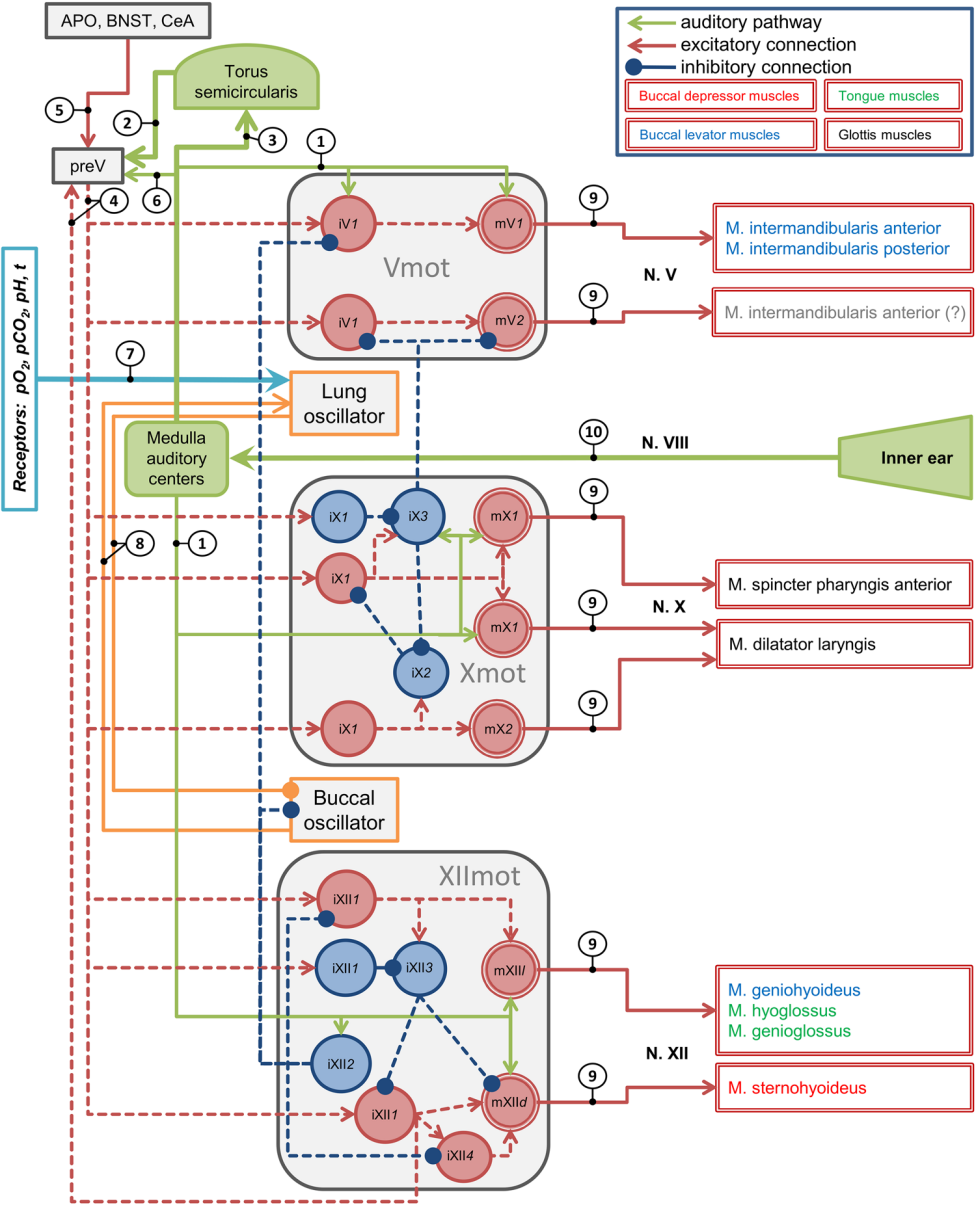
Hypothetical connection pattern of audio–vocal integration based on electrophysiological responses of motor neurons (twin circles) and interneuron types (circles, neuron types labeled according to Figure 3) in the medulla oblongata of *Bombina orientalis*. The broken lines represent hypothetical connections based on neuron types and their timing of AP generation as shown in this study. Solid lines indicate connections based on literature cited by numbers. Fast medullary input and midbrain input from torus semicircularis (green) activate premotor interneurons and motor neurons as discussed in the main text. This leads to short‐latency activation of depressor motor neurons (mXII*d*), while activity of levator motor neurons is initially suppressed via inhibitory interneurons (red) in *phase A*. During *phase B*, depressor activity is terminated by delayed firing inhibitor interneurons. Simultaneously, levator units (mV*1*, mX*1*, mXII*l*) are depolarized by excitatory interneurons. Vmot, motor nucleus of N. V (vagal nerve); Xmot, motor nucleus of N. X (glossopharyngeal nerve); XIImot, motor nucleus of N. XII (hypoglossal nerve). (1) Connections of auditory centers into motor neuron and interneuron types were demonstrated by N. XIII stimulation in this study. (2) Efferents of the torus semicircularis feed into premotor centers such as the pretrigeminal nucleus (preV; Walkowiak 1992; Walkowiak and Luksch 1994). (3) Auditory efferents of the torus semicircularis feed into motor centers such as trigeminal and vagal motor nuclei in the archaeobatrachian frogs *B. orientalis* and *D. pictus* (Walkowiak 1992; Strake et al. 1994; Walkowiak and Luksch 1994). (4) The activation of premotor centers such as the preV (Schmidt 1992; Zornik and Kelley 2008) via or directly by the torus semicircularis (Luksch and Walkowiak 1998) may be necessary to coordinate the activity of the different motor units involved selectively. The anuran preV and the motor nuclei are connected reciprocally (Schmidt 1992; Zornik and Kelley 2007). (5) Higher brain centers are involved in the regulation of call behavior as well. Electrical stimulation and lesion experiments, as well as studies using testosterone implants in frogs (*R. pipiens*, *Hyla arborea*), identified the hypothalamic anterior preoptic area (APO) as a crucial component for the initiation of mate vocal behavior (Schmidt 1968, 1973, 1984; Knorr 1976; Wada and Gorbman 1977a, 1977b). This area was interpreted to be a command region of frog vocalization (Gerhardt and Huber 2002). Moreover, the central amygdala (CeA) initiates vocal responses to communication signals, and the neighboring bed nucleus of the stria terminalis (BNST, nucleus striae terminalis) plays a role in the initiation of vocalizations (Hall et al. 2013; Kelley et al. 2020; Kelley 2022). (6) A route from the medullary auditory centers (i.e., the superior olive) to the motor neurons via the preV was hypothesized by Aitken and Capranica (1984; see main text). (7) The input of receptors for blood gases (pO_2_, pCO_2_), pH, and temperature (t; reviewed by Milsom et al. [2022]). (8) The potential inhibitory input into the oscillator for buccal and lung ventilations was included (see discussion). Further connections of these oscillators were omitted for the sake of clarity. (9) Connections proposed by Huggenberger and Walkowiak (2024). The question mark (?) indicates the speculative activation of the anterior intermandibular muscle that may close the nares (Huggenberger and Walkowiak 2024). (10) The auditory pathway in anurans was reviewed by Capranica (1976), Wilczynski and Capranica (1984), and Wilczynski and Endepols (2007).

In more detail, the hypothetical network may look like the one described in Figure 8, which is based on the data from the literature discussed above, as well as the firing patterns of the neuron types defined in this study. The first step is the activation of the motor network of Vmot, Xmot, and XIImot by auditory input. The next step is the motor pattern generation.

#### 4.3.1 Auditory Activation

Some medullary interneurons and numerous motor neurons in the areas of the motor nuclei of Nn. V, X, and XII (Vmot, Xmot, and XIImot) get fast excitatory auditory input (“fast EPSPs”) with latencies shorter than 10 ms (Figure 3). This indicates that audio–vocal integration does not exclusively involve higher brain centers such as the torus semicircularis (Luksch and Walkowiak 1998; Penna et al. 2023) but also centers within the medulla oblongata (Figure 8). The mean latency of EPSP onset in the torus semicircularis in *B. orientalis* after stimulation of the auditory branchlet of N. VIII was found to be 20 ms (Walkowiak 1980; Luksch and Walkowiak 1998; Endepols and Walkowiak 2001). The mean latency of neural reaction onset in the superior olive of *D. pictus*, a related archeobatrachian species, was found to be 7.6 ± 4.3 ms (Luksch and Walkowiak 1998) and is thus similar to the “fast AP” latency of 9.9 ± 2.3 ms in our N. XII motor neurons (mXII*d*). The very short latencies of most of these mXII*d* neurons (Figure 3) suggest that these neurons get input from neurons of medullary auditory centers such as the dorsal medullary nucleus or the superior olive (Wilczynski and Endepols 2007). Additionally, other motor neurons such as mV*1*, mX*1*, and mXII*l* get fast excitatory input but were inhibited thereafter (Figure 3). Thus, it is likely that the same neural path leading to this fast excitatory input of motor neurons also activates inhibitory interneurons (e.g., iX*3*, iXII*2*; Figure 3b) simultaneously, which may, in turn, inhibit transiently the fast motor neuron excitation (Figure 8). However, the unsteadiness of AP generation by the “fast EPSP” suggests that this input is not strong.

Because there are neurons in the superior olive with latencies shorter than 5 ms (Luksch and Walkowiak 1998), a route from the superior olive to the motor neurons via the preV, as hypothesized by Aitken and Capranica (1984), is also consistent with our data (Figure 8). However, in *B. orientalis*, a pathway from the dorsal acoustic nucleus in the medulla oblongata via the reticular formation to mXII*d* is more likely because we found interneurons with extremely short latencies in the area of Xmot and XIImot (<5 ms; see above). These fast responses are in the range of AP latencies of neurons described in the mammalian caudal pontine reticular nucleus (PnC), which are known to mediate the startle response (Lingenhöhl and Friauf 1992, 1994). Thus, one can speculate that the acoustic startle response in mammals, which is mediated via the cochlear nucleus (cochlear root neurons) and the caudal PnC (reviewed in Zheng and Schmid 2023), may have its evolutionary origin in this fast motor input of anuran amphibians. Hale et al. (2002) concluded that, in fish, the startle neural circuit itself is not conserved. In larval and adult anurans, the acoustic startle response is mediated by giant medullary neurons (Mauthner cells; Will 1991), which derive from rhombomere 4 (reviewed in Korn and Faber [2005]). The mammalian PnC, so far only described for humans, macaques, rats, and mice, is a derivative of rhombomere 7 (Schröder et al. 2023). The difference in rhombomeric origin makes the homology of Mauthner cells and giant PnC cells, which project to motor neurons (Zheng and Schmid 2023), unlikely. However, Mauthner cells seem to be absent in *Bombina* and *Discoglossus* species (Will 1986, 1991).

The central rhythm generator for lung oscillations is located in the rostral brainstem (McLean et al. 1995; Perry et al. 1995). There is evidence that the rhythmic pattern of respiration in all tetrapods is mediated by two coupled oscillators (Wilson et al. 2002). In *Rana catesbeiana*, two regions within the ventral medullary reticular formation have been shown to be crucial for the generation of respiratory patterns. The lung oscillator is situated rostral to the root of N. X and the buccal oscillator caudal to N. X (Wilson et al. 2002, 2006; Gargaglioni and Milsom 2007). Although the buccal oscillator was able to mediate the rhythm of buccal oscillation by its own, it was shown that both oscillators are required to generate lung ventilation (Vasilakos et al. 2006). The buccal oscillator may activate the lung oscillator, while the latter inhibits the buccal oscillator (Wilson et al. 2006). Thus, there is evidence for two coupled oscillators in frogs associated with the buccal expansion and compression (Baghdadwala et al. 2015, 2016). It has been suggested that the caudal buccal oscillators act to bring the lung oscillator(s) to threshold and establish its (their) rhythm (Milsom et al. 2022).

In the context of this proposed coupled oscillator hypothesis (Milsom et al. 2022), it is not clear how additional vocal movements, such as the movements of the laryngeal structures, are coordinated with pulmonal respiration. Here, the question arises as to where and how auditory input triggers vocal generation and inhibits buccal oscillations and voiceless lung ventilation. A possible mechanism could be that interneurons with short latencies (iXII*2*) inhibit the buccal oscillator, which would then activate the lung oscillator (Figure 8).

We found the latencies of depressor motor neurons of XIImot (mXII*d*) and interneurons of Xmot and XIImot (iX*3*, iXII*2*) more than 60 ms shorter than the latencies of fast APs of the other motor neurons, mV*1* and mXII*l*. These extremely short latencies in mXII*d*, iX*3*, and iXII*2* (Figure 3) together with the strong activation of the N. XII early in *phase A* suggest that the XIImot area plays a crucial role in initiating lung inflation and vocalization and may function as a supplementary interface of audio–vocal integration (Figure 8). The caudal position of this interface may be useful since the mXII*d* neurons and related interneurons are activated in advance of other motor units during call generation. This potential supplementary interface is moreover topographically related to an area identified as the dominant buccal oscillator, located caudally of the N. X root in the reticular formation, while the lung oscillator is situated further rostrally within the brainstem (Wilson et al. 2002; Baghdadwala et al. 2015; Milsom et al. 2022). Thus, this potential supplementary interface at XIImot may first inhibit the buccal oscillator (Figure 8) before lung inflation is elicited so that it would not inhibit the lung oscillator, in turn (Wilson et al. 2006).

The extremely fast but weak excitatory input to most mXII*d* neurons—as judged by the low number of fast APs—may not suffice to elicit fictive lung ventilation after N. VIII stimulations. The activation of higher auditory and premotor centers such as the torus semicircularis (Luksch and Walkowiak 1998) and the preV (Schmidt 1992; Zornik and Kelley 2008) may be necessary to coordinate the activity of the different motor units involved selectively (Figure 8). Accordingly, it is known that the preV and the motor nuclei are connected reciprocally (Schmidt 1992; Zornik and Kelley 2007) so that the activation of the preV via this potential supplementary interface at XIImot (potentially via iXII*1* neurons) may be crucial to coordinate neural patterns for vocalization (Figure 8). However, this hypothetical connection needs to be validated in future studies.

#### 4.3.2 Vocal Motor Pattern Generation

The fast auditory input (shown in this study), input from the preV (Schmidt 1992; Zornik and Kelley 2007), and midbrain input from torus semicircularis (Luksch and Walkowiak 1998) seem to activate together the premotor and motor neurons. According to our hypothetical neural network (Figure 8), this activation pattern leads to short‐latency activation of depressor motor neurons (mXII*d*), while activity of levator motor neurons is initially suppressed, probably via fast inhibitory interneurons in *phase A*, for example, by iXII*2* neurons (Figure 3b).

During *phase B*, depressor activity is terminated by slightly delayed firing of inhibitory interneurons (Figure 8), such as iXII*3* neurons (Figure 3b). In parallel, the potential motoneurons responsible for the closure of the nares (mV*2*) may be activated by iX*3* neurons. Simultaneously, levator units (mV*1*, mXII*l*) are depolarized by excitatory interneurons (Figure 8), such as iV*1* or iXII*1* neurons (Figure 3b). These levator subnetworks may be delayed by fast inhibitory interneurons such as iXII*2* (Figure 8). In this regard, it is important to note that we cannot characterize our interneuron types summarized in Figure 3b as inhibitory or excitatory. We defined them by plausibility as inhibitory or excitatory within our hypothetical network (Figure 8), which hopefully can be used as a working model in future research.

The iX*1* neurons may activate the motoneurons of N. X in *phase B*. These mX*1* neurons may innervate the muscles operating the larynx. The M. dilatator laryngis opens the glottis, and the M. sphincter pharyngis anterior can close it (Lörcher 1969; Schneider 1970). However, there is no information on which muscle is activated by these motoneurons in *B. orientalis*. For example, the activity pattern of the two larynx muscles mentioned during respiration is not consistent in different neobatrachian species (*R. pipiens* [DeJongh and Gans 1969] and *R. catesbeiana* [West and Jones 1975]). Although the glottis must be open during *phase B* (M. dilatator laryngis) for inspiration and vocalization, it is possible that the sphincter muscle may control the tension of the vocal folds in parallel. This topic needs further investigation.

According to our hypothetical network (Figure 8), at least some of the mX*1* neurons innervate the dilatator laryngis muscle as well as the mX*2* neurons (see above). Whether these two neuron types (mX*1*, mX*2*) correspond to the two different motoneuron types in the bullfrog *R. catesbeiana* separated by different intrinsic AP firing patterns (Zubov et al. 2021) is a matter of speculation. The firing pattern, that is, the frequency of APs, of these neurons in our whole brain preparation did not show any differences, pointing to two distinct neuron type populations other than their classification for *phases A* and *B*, respectively (Figure 4).

The activation of mX*1* neurons and their interneurons iX*1* may be delayed in order to fire in *phase B* by inhibitory interneurons such as iX*2* (Figure 3b), which are, in turn, inhibited in *phase B* via iX*3* neurons (Figure 8). The latter (iX*3*) may also inhibit the small motoneuron population mV*2* for the closure of the nares.

In *phase C*, depressor units (mXII*d*) may be reactivated by interneurons that were inhibited during *phases A* and *B* (iXII*4*; Figure 8).

So far, the function of inhibited iV*2* neurons (Figure 3b) is not clear for the hypothetical network in Figure 8; thus, they were not included. It may be possible that the decaying inhibition of this rare interneuron type facilitates the activity of potential upstream neurons such as mV*2* or iXII*4*.

The hypothetical network in Figure 8 derives from the pooled data of male and female brains. This pooling may be a potential source of error because only male *B. orientalis* are known to utter advertisement calls. However, we could show recently that probably the same central pattern generator produces fictive vocalizations as well as fictive respiration in isolated brains of both sexes (Huggenberger and Walkowiak 2024).

## 5 Conclusion

The hypothetical network in Figure 8 is a scheme to illustrate the combined network of respiration and vocalization in anurans and its auditory input. Our findings (Figure 3) and interpretations (Figure 8) indicate that audio–motor integration in anurans is not strictly hierarchical because a preceding pathway parallels the main audio–motor interface, that is, the torus semicircularis (Luksch and Walkowiak 1998; Wilczynski and Ryan 2010), at the level of the medulla oblongata. The mechanism of lung ventilation and auditory‐elicited vocalization makes the stem anuran *Bombina* spec. an attractive model to study the coordination of its joint neural network on the cellular level in air‐breathing vertebrates.

## Author Contributions

**Stefan Huggenberger**: data analysis, investigation, methodology, data curation, visualization, writing – original draft and editing. **Wolfgang Walkowiak**: conceptualization, data curation, formal analysis, investigation, methodology, validation, project administration, resources, supervision, validation, writing – review and editing.

## Conflicts of Interest

The authors declare no conflicts of interest.

## Peer Review

The peer review history for this article is available at https://publons.com/publon/10.1002/cne.70088.

## Data Availability

All data that support the findings of this study are available from the corresponding author.
